# Rotating Shift-Work as an Independent Risk Factor for Overweight Italian Workers: A Cross-Sectional Study

**DOI:** 10.1371/journal.pone.0063289

**Published:** 2013-05-10

**Authors:** Pamela Barbadoro, Lory Santarelli, Nicola Croce, Massimo Bracci, Daniela Vincitorio, Emilia Prospero, Andrea Minelli

**Affiliations:** 1 Department of Biomedical Science and Public Health, School of Medicine—Università Politecnica delle Marche, Ancona, Italy; 2 Department of Molecular and Clinical Sciences, School of Medicine—Università Politecnica delle Marche, Ancona, Italy; 3 Department of Earth, Life and Environmental Sciences, University of Urbino Carlo Bo, Urbino, Italy; College of Pharmacy, University of Florida, United States of America

## Abstract

**Background:**

A job-related factor is attracting a growing interest as a possible determinant of body weight gain in shift-workers.

**Objective:**

The aim of the study was to reinvestigate the issue of overweight between rotating shift workers and daytime workers, taking into consideration possible confounding covariate factors.

**Methods:**

This is a cross-sectional study, conducted by reviewing data from subjects participating in an occupational surveillance program in 2008. Participants answered a self-administered questionnaire to retrieve information about socio-demographic factors and working conditions (job schedule type, job-related physical activity, time in job), subjective health status, health care visits during the previous year, and lifestyle factors (dietary habits, leisure time physical activity, alcohol consumption). Participants underwent a medical examination for measurement of BMI, and acquisition of medical history.

**Results:**

Compared to daytime workers (N = 229), rotating shift workers (N = 110) displayed higher BMI (mean BMI was 27.6±3.9 and 26.7±3.6 for shift workers, and daytime workers, respectively; p<0.05). Logistic regression analysis allowed to highlight the role of rotating shift-work as an independent risk factor for increased body weight (OR 1.93, 95%CI 1.01–3.71), being aged between 35 and 54 years was a major determinant of increased BMI (OR 2.39, 95%CI 1.14–5.00). In addition, family history of obesity was the strongest determinant of overweight/obesity (OR 9.79, 95%CI 1.28–74.74). Interestingly, no significant association was found between overweight and other potentially relevant factors, such as diet quality and food choices, alcohol consumption, levels of occupational and leisure-time physical activity.

**Conclusions:**

Present findings seem to support the notion that rotating shift work is an independent risk factor for overweight, regardless of workers' dietary habits and physical activity levels.

## Introduction

Overweight and obesity are major healthcare problems in today's societies. In fact, excessive body weight accumulation can lead to an elevated risk of some types of cancer, cardiovascular and metabolic illnesses, osteoarticular diseases, and to an increase of all-cause mortality risk [1], [2]. Multiple endogenous, environmental and life-style behavioral factors have been variably associated to the worldwide soaring prevalence of overweight [3]; although the pathways are unclear, a job-related factor attracting growing interest as a possible determinant of body weight gain and related chronic diseases is the protracted exposure to shift-work [4], [5].

Working in shifts is becoming a mighty common for an increasing proportion of Western population. In fact, the number of jobs including shift work schedules and irregular working hours has massively grown during the last decades, and nowadays about 10%–20% of the workforce in Europe and US is exposed to night work schedules [6], [7]. In 2004, a study by Eurispes has calculated that, in Italy, over two and a half million people - approximately 15–20% of the Italian workforce - are employed in shifts from 22:00 to 6:00 am [8].

Large amount of research has been published concerning the health effects of shift-work [9]–[12]. Shift-work has been related to disrupted sleep/wake cycle and chronic desynchronization between endogenous circadian rhythms and behavioral cycles, thereby leading to endocrine and metabolic alterations, such as hypertension, glucose intolerance and lipid profile disturbances [13], [14]. In addition, shift workers have been shown more frequently to adopt unhealthy behaviors, such as smoking [15], incorrect feeding behavior with fragmented eating patterns and eating at night [16], [17], physical inactivity [18]. All these factors may synergically contribute to promoting an increase in body weight and Body Mass Index (BMI).

In recent years, several cross-sectional and longitudinal studies have investigated the association between shift-work and body weight changes, yielding varying results. While cross-sectional studies have consistently demonstrated greater body weight and higher obesity rate among shift workers than in daytime workers [19], [20], [21], [22], longitudinal studies, although showing clear evidence for a crude positive association, seem to bring insufficient evidence when potential confounders were considered [4]. Differences in job typologies, in patterns of shift-work exposure, in ethnic and socio-demographic features of worker populations examined in the various studies, as well as the heterogeneous nature of confounding covariates taken into account, have probably hampered the achievement of a conclusive evidence on the issue.

In the present cross-sectional study, we intended to reinvestigate the issue of overweight in adult workers; in a population of Italian railway service male employees, we searched for differences in BMI between rotating shift workers and daytime workers, taking into consideration possible confounding covariate factors. Preliminary results were previously reported [23].

## Methods

### Design and sample

This is a cross-sectional study, conducted by reviewing data from subjects participating to an occupational surveillance program in 2008. Consecutive workers were asked to participate in the study. Based on prior research on prevalence of overweight and obesity in Italy, we expected an overall proportion of 47% persons in these categories [24], a total sample size of N = 300 (with 2∶1 ratio of groups) was calculated a priori to detect an increase of overweight obesity of 33%, with a power ≥0.80 and α = 0.05 (two-tailed). The theoretical sample size has been increased by 15% in order to include a satisfactory final number of participants. Of the 353 eligible workers, 345 (97.7%) agreed to participate in the study. Given the occurrence of six incomplete/unclear questionnaires; the final study sample included 339 male subjects (229 daytime workers and 110 shift workers), all employed in the Italian railway service in central Italy. Participants answered a self-administered questionnaire and underwent a medical examination for measurement of clinical data. The study was conducted in accordance with the Declaration of Helsinki's ethical standards. Being part of the standard occupational health surveillance it needed no formal approval by the local ethics committee (ASUR, Area Vasta 2), which was nevertheless consulted and which granted an informal authorization. Subjects gave their written informed consent to participate in the study.

The job schedule type was divided into rotating shift work and daytime work. The morning shift started at 0600 h and continued to 1400 h, the afternoon shift started at 1400 h until 2200 h, and the night shift started at 2200 h until 0600 h. All rotating shift workers worked continuously (including week end) 2 days in the morning shift, 2 days in the evening shift, and 2 days in the night shift, with 3 days of rest after the night shift. Daytime workers worked 8 h/day, 5 days a week starting between 0700 h and 0800 h. Workers were occupied in different jobs: technical work, office work, fireman, ticket inspector, engine-driver, railway station personnel.

A “case” was defined as a worker characterized by a BMI>24.9; a “control”, was defined as a worker with a BMI lower than 24.9.

### Measurements

A self-administered questionnaire was constructed to retrieve information about socio-demographic factors and working conditions, subjective health status, health care visits, and lifestyle factors. The sections of the questionnaire are described below.

#### Socio-demographic factors and working conditions

These factors included age, type of occupation at the time of the survey, and occupational physical activity.

#### Lifestyle factors

These factors included diet, alcohol consumption, and leisure time physical activity. An Italian version of the Rate Your Plate Eating Pattern Assessment (RYP) was used to assess fat intake, and to provide qualitative nutrition information related to typical food choices of participants [25]. Alcohol consumption was assessed according to a graduate frequency test. Average daily consumption per person was classified as follows: Class I, 0–40 grams; Class II, 41–60 grams and Class III, >60 grams [26].

#### Physical activity

Leisure-time physical activity has been assessed by the Minnesota questionnaire [27]. Mean daily energy expenditure (MAI), expressed in kcal/day was computed by summing the products of the metabolic cost of each activity, its average duration, and the number of occasions across the 12-month period, and then dividing by 365. The natural logarithm of (MAI) was used in all regression analyses due to the skewness of the untransformed variable. Moreover, the Tecumseh Self-Administered Questionnaire was used to estimate occupational physical activity [28]. Given the results of the questionnaire, workplace physical activity was classified into: mild, moderate and heavy. Subjects were given detailed instructions for completing the questionnaires and both questionnaires were administered by the same investigator (DV).

#### Clinical risk factors

A detailed medical anamnesis including information about personal and family history of obesity was retrieved. Moreover, measurement of Body Mass Index (BMI) was obtained. Participants' BMI was classified into the following categories: normal weight (<24.9 kg/m^2^); overweight (>25.0–29.9 kg/m^2^). The reliability of the clinical measurements was assured by using the same observers, environment, and instruments during health examinations.

### Validation of questionnaire

The questionnaire was pre-tested using a random sample of participants. Concurrent construct validity was estimated by comparing specifically designed items within the instrument with other items measuring the same concept. For example, we evaluated the association between participants rating their health as good-very good and those who declared not feeling healthy in the last month (chi-square<0.001).The reliability coefficient for dichotomous variables (tested by using the Kuder-Richardson formula 20 test) was 0.719; and for Likert-scale items (tested by using Cronbach's alpha) was greater than 0.7.

Frequency and percentage distributions were used to describe the data. The statistical differences between the groups of shift/non-shift workers were analyzed by chi-square test.

Multiple logistic regression analysis was performed to identify the variables associated to overweight (BMI>24.9). Covariates were included into the models with a stepwise procedure. Explanatory variables that were associated with the outcome at a significance of 0.20 or less at univariate analysis, were included as independent variables to adjust for the indirect effects of other variables. Association between the characteristics and overweight was expressed as odds ratios (ORs) and 95% confidence intervals (CIs). Standard post-estimation tests were used to assess the models validity, i.e. F-statistics and ROC curves observation. Level of significance was set at 0.05. Analyses were performed using STATA, version 9 (Stata Corp., College Station, TX, 2005).

## Results

Group characteristics are shown in Table 1. Compared to daytime workers, rotating shift workers: i) displayed higher mean BMI value (27.6±3.9 and 26.7±3.6 for shift workers and day workers, respectively; p<0.05) and higher percentage of overweight/obese subjects; ii) were significantly older (mean age 48.6±8.3 and 42.1±12.2 for shift workers and day workers, respectively, p<0.05); ii) were less physically active at the workplace, with 7.3% of shift workers vs 53.7% of day workers reporting “heavy” loads of occupational activity, but were significantly more active during leisure-time (MAI index was 181.5±173.9 Kcal/day in daytime workers vs 214.2±221.6 Kcal/day in shift workers, p<0.05); iii) had worse quality of food intake, as evidenced by lower RYP score (36.1±5.8 in shift workers vs 37.7±5.6 in day workers, p<0.05), and consumed alcohol less frequently. No significant differences in family history of obesity were found between the two groups.

**Table 1:** Distribution of workers by selected characteristics.

	*Daytime workers*	*Shift workers*	*P value*
	*(n = 229)*	*(n = 110)*	
***Age***			
<35 years	15.7%	5.45%	<0.001
35–54 years	72.9%	66.4%	
≥55 years	11.4%	28.2%	
***Weight***			
Underweight/normal weight	32.7%	20.9%21.9	0.024
Overweight or Obese	67.3%	79.1%	
***Occupational physical activity***			
Mild	32.3%	77.3%	<0.001
Moderate	14.0%	15.5%	
Heavy	53.7%	7.3%	
***Leisure time Physical activity (MAI)***	181.5±173.9	214.2±221.6	0.155
***Rate Your Plate score***	37.7±5.6	36.1±5.8	0.015
***Alcohol consumption***			
<40 grams/day	92.0%	96.2%	0.139
41–60 grams/day	5.4%	3.8%	
>60 grams/day	2.7%	0.0%	
***Family history of obesity***	6.1%	10.9%	0.120

Logistic regression analysis (see Table 2) allowed to highlight the role of rotating shift-work as an independent risk factor for increased body weight (OR 1.93, 95%CI 1.01–3.71), after adjusting for selected confounding covariates (such as age, family history of obesity, alcohol consumption, and physical activity). The age of participants also resulted as a factor independently associated to overweight: in particular, being aged between 35 and 54 years was a major determinant of increased BMI (OR 2.39, 95%CI 1.14–5.00), whereas the association was not significant at older age (OR 1.76, 95%CI 0.67–4.60). In addition, family history of obesity appeared as the strongest determinant of overweight/obesity in participating workers (OR 9.79, 95%CI 1.28–74.74). Interestingly, no significant association was found between overweight and other potentially relevant factors, such as diet quality and food choices, alcohol consumption, levels of occupational and leisure-time physical activity (see Table 2).

**Table 2:** Factors associated to overweight in workers at multivariate modeling.

	OR	95% CI	p-value
***Age***			
<35 years	1		
35–54 years	2.38	1.13–5.00	0.021
≥55 years	1.76	0.67–4.60	0.252
***Rotating Shift-work***			
No	1		
Yes	1.93	1.01–3.71	0.048
***Family Hystory of obesity***			
No	1		
Yes	9.79	1.28–74.74	0.028
***Alcohol consumption score***			
<40 grams/day	1		
41–60 grams/day	0.74	0.23–2.37	0.606
>60 grams/day	3.84	0.42–35.38	0.235
***Rate Your Plate*** ** score**	1.08	0.98–1.19	0.104
***Occupational physical activity***			
Mild	1		
Moderate	0.99	0.44–2.19	0.972
Heavy	0.85	0.45–1.60	0.608
***Leisure time physical activity***			
No	1		
Yes	1.00	1.00–1.01	0.667

## Discussion

This is a cross-sectional study aimed at investigating, on a representative sample of male workers employed in Central Italy, the association of shift-work and other covariate factors to body weight. Crude results showed that our rotating shift workers display higher prevalence of overweight compared to daytime workers; in addition, evidence from multivariate analysis pointed to shift-work as an independent risk factor for being overweight, after adjusting for selected potential confounders, such as age, diet quality, alcohol consumption, levels of occupational and leisure-time physical activity, and family history of obesity.

Present findings, that extend previous, preliminary observational evidence gathered by our group [23], are in good agreement with reports from recent cross-sectional studies conducted on diverse worker populations across different countries. Sookoian and colleagues [19], in Argentina, reported that male factory workers involved in rotating shift schedules have higher BMI and increased OR for metabolic syndrome compared to day workers, independently of age and physical activity; Zhao and colleagues [20] found that shift-work is independently associated to overweight and obesity in Australian female nurses, after adjusting for diet quality, smoking status and physical activity levels; Manenschijn and colleagues [21], in the Netherlands, reported increased BMI in male rotating shift workers employed in a textile factory; Macagnan and colleagues [22] documented the independent contribution of night shift-work to overweight and abdominal obesity among Brazilian factory workers of both genders. More conflicting evidence emerge from longitudinal studies; although a crude relationship between shift-work and body weight changes has been consistently reported, such relation was not always confirmed when relevant confounders were taken into account. In a retrospective study on Italian male workers employed in a municipal enterprise for street cleaning and waste collection [15], night work was associated to higher BMI and more risky lipidemic profile; two prospective studies on Japanese male blue-collars found larger BMI increase in shift workers, relative to daytime workers, after 1 year [16] and 10 years follow-up [29]; more recently, Zhao and colleagues [30] reported that Australian female nurses that changed from shift- to day-work schedules decreased their BMI over follow-up period, whereas shift-work maintainers and day-to-shift changers increased their BMI. Different, inconsistent results were instead reported by other studies: no significant effect of night work on body weight changes was found after 5 years follow-up in a group of French female nurses [31]; even more surprisingly, results pointing to the opposite direction were reported on a mixed population of nurses and other employees, with shift workers losing - and day workers gaining - body weight and BMI [32].

In our study, family history of obesity emerged as the major determinant for body weight increase, thus emphasizing the importance of heritable tracts and trans-generational aspects in influencing the risk of overweight in adult workers. This finding is not surprising. Twin and adoption studies reported high heritability of adiposity [33], [34], thus pointing to a relevant genetic component in overweight and obesity [35]. In addition, it is well established that physical, relational and social characteristics of the family environment contribute to shaping children's dietary intake and eating habits [36], as well as their involvement in physical activity [37], thereby inducing food preferences and behavioral patterns that usually persist in adulthood [38] and promoting long-term effects on body weight trajectories. In line with present findings, parental overweight was found to be associated to higher BMI and abdominal obesity in a population of male and female workers of a poultry processing plant in Brazil [22].

Another important covariate is age. Being relatively young, i.e. between 35 and 54 years of age, appeared as an independent risk factor for weight gain in our sample, while older age (≥55) was non-significantly associated to BMI changes. These results are in line with previous evidence showing that male shift workers have higher proportion of abdominal obesity [39] and increased BMI and cortisol levels [21] compared to day workers, with differences that are significant only in workers aged 50 years or younger, but not at older age. Macagnan and colleagues [22] showed that the risk for overweight is linearly associated to increasing age, but their sample included workers not older than 50 years. Collectively, present and previous observations suggest that shift-work may play an age-dependent role in determining the risk for overweight in adult workers, with effects that are possibly moderated over life time. The cross-sectional nature of our study hampers a causal explanation of this finding. Possibly, older subjects somehow learn to adjust to shift-work, thus habituating to stress and sleep cycle disturbances; alternatively, there may be a selection bias, with individuals who cannot adjust to night work stopping to work in shifts at younger age.

Eating behavior, energy intake and dietary choices are all factors that can influence body weight, and represent potential confounders in our study. As a dietary assessment instrument we used the RYP test, a self-administered food-frequency questionnaire whose validity was previously established in calibration studies through correlation coefficients relating RPY score and various measures of dietary fat, saturated fat and cholesterol intake [25], and that was successfully used in a variety of settings and demographic groups [25], [40], [41]. Present findings show that the mean total RPY score in daytime workers was significantly, though slightly, higher than that reported by rotating shift workers; however, multinomial regression analysis reveals that quality of nutrition is not an independent factor in determining the risk for overweight in our sample. Most previous studies found only small differences in total energy/macronutrient intake and eating habits between daytime and shift workers [42]–[44], thus suggesting that food intake itself cannot fully explain possible body weight gain as a result of shift-work. However, further aspects need to be considered. First, work schedules exposing humans to light during night hours can promote irregular food intake patterns [14]; this particular aspect is in fact poorly assessed by RYP test. In addition, shift-work is generally associated with chronic misalignment between endogenous circadian timing system and behavioral cycles, with adverse metabolic consequences [21], [45]; shifting the time of food intake is reported to affect postprandial glucose and insulin levels [19], [39], [46], and increase body mass [47]. Taken collectively, these observations suggest not to withdraw attention towards diet-related aspects of shift work, and point instead to a possible role of education programs on eating behavior as preventive strategies in this group of workers.

As for alcohol consumption, mild differences were observed in the present study between daytime and rotating shift workers; however, we found that drinking habits did not significantly affect OR for being overweight. Though seemingly paradoxical, this finding seems rather consistent with previous literature. In fact, although ethanol consumption is usually assumed to be a risk factor for body weight gain, the relationship between weight and alcohol intake is unclear, with some epidemiological studies reporting a positive association, others a negative association, and others no relationship at all [48]. Interestingly, a longitudinal study conducted in male Japanese workers showed that drinking habits actually decreased the risk of overweight [16].

Physical activity is regarded as another important factor influencing body weight and metabolism. Previous literature stating that employees involved in shift-work schedules tend to become physically less active, since having less time to participate in organized, social sporting activities [18], have suggested that physical activity should be regarded as an intermediate factor, rather than a confounder, in the relationship between shift-work and overweight. In our sample, bivariate analysis revealed that in rotating shift workers, compared to daytime workers, levels of physical activity were lower at the workplace, but with a trend to be higher during leisure-time; in fact, rotating shift workers seem to compensate for their lower occupational activity with more physical activity in leisure-time. Similarly, a recent study conducted in a large population of Australian workers reported that individuals with mostly sitting jobs were more likely to be sufficiently active during leisure-time, as compared to workers with mostly walking and heavy labor jobs [49]. All together, these observations suggest to consider physical activity as a confounding covariate. Our logistic regression analysis revealed that physical activity levels, either at the workplace or during leisure-time, were not independently associated to body weight changes. These findings are only partially in line with previous evidence, which is rather conflicting. The association between occupational physical activity and weight gain in adult workers is still object of debate, with only five out of ten cross-sectional studies finding evidence for a positive association [50]. In contrast with our results, a positive association of leisure-time physical activity with obesity risk in working adults has been recently reported [49].

Some limitations of the present study need to be pointed out. First, the sample size is relatively small; in addition, the remarkable homogeneity of study participants – only male workers, sharing similar socio-economic characteristics and level of occupation – and the uniformity of rotating shift-work schedules at which shift workers were exposed, although probably enhancing the quality of questionnaire data, likely reduced the generalizability of our data to other worker populations. Second, some potential confounders have not been considered in our study, such as the cumulative number of years in shift-work, smoking habits, sleep quality and stress symptoms, all factors that may indeed have a role in influencing body metabolism and tolerance to shift-work. Third, here we chose to use overweight instead of obesity as the study outcome because our main assumption was based on a public health perspective rather than a clinical one. Interestingly, when running the analysis with obesity as the outcome, we found no differences between rotating shift and daytime workers. However, the validity of choosing overweight as preclinical outcome gains support from a recent prospective study showing that an extended period of rotating shift work is associated with an increased risk of type II diabetes in women, which is mainly mediated through BMI and body weight changes [44].

In conclusion, present findings seem to support the notion that rotating shift workers, regardless of their dietary habits and physical activity levels, may tend to accumulate excessive body weight through pathophysiological mechanisms that are, at least in part, strictly dependent on job-related variables and working time schedules. Other important risk factors for becoming overweight are parental overweight and relatively young age. The present results evidence shift work as an independent risk factor for overweight/obesity among male workers; the results should not to be overlooked, especially because the analysis of risk has been adjusted for others modifiable lifestyle factors (such as, occupational and leisure time physical activity, nutritional habits, alcohol consumption, and occurrence of overweight/obesity in the family of origin). Policy makers involved in planning production strategies, and workforce organization should, therefore, take this findings in serious consideration. If confirmed in other study populations, this supportive evidence implies that the reduction of shift work (at least when possible) could help improving health and wellbeing among working population.
