# Correction: Rotating Shift-Work as an Independent Risk Factor for Overweight in Italian Workers: A Cross-Sectional Study

**DOI:** 10.1371/annotation/2f0fb30e-1db9-4d28-97aa-eaf0a55cfa2c

**Published:** 2013-06-19

**Authors:** Pamela Barbadoro, Lory Santarelli, Nicola Croce, Massimo Bracci, Daniela Vincitorio, Emilia Prospero, Andrea Minelli

There is an error in the article title. The correct title is:

Rotating Shift-Work as an Independent Risk Factor for Overweight in Italian Workers: A Cross-Sectional Study 

